# Late gestational nutrient restriction decreases placental size and calf birth weight without altering uterine blood flow in primiparous beef females

**DOI:** 10.1093/jas/skaf163

**Published:** 2025-05-13

**Authors:** Colby A Redifer, Abigail R Rathert-Williams, Allison M Meyer

**Affiliations:** Division of Animal Sciences, University of Missouri, Columbia, MO 65211, USA; Division of Animal Sciences, University of Missouri, Columbia, MO 65211, USA; Division of Animal Sciences, University of Missouri, Columbia, MO 65211, USA

**Keywords:** beef heifer, developmental programming, Doppler ultrasonography, fetal growth, pregnancy, uteroplacenta

## Abstract

To investigate impacts of late gestational nutrient restriction in first-parity beef females on prenatal nutrient availability to calves, fall-calving heifers [body weight (**BW**): 472 ± 33 (SD) kg; body condition score (**BCS**): 5.4 ± 0.5] were individually-fed 100% (control; **CON**; *n* = 13) or 70% (nutrient restricted; **NR**; *n* = 13) of metabolizable energy and metabolizable protein requirements for maintenance, pregnancy, and growth from day 160 of gestation to calving. Maternal measures were determined every 21 d (BW and metabolites) or 42 d (BCS, backfat, and longissimus muscle area) during gestation and post-calving. Doppler ultrasonography of both uterine arteries was conducted every 21 d until day 244 of gestation. At birth, calf BW and size were measured, and expelled placentas were dissected and dried. Targeted messenger ribonucleic acid (**mRNA**) expression was determined for cotyledons. Data were analyzed with nutritional plane, treatment initiation date, heifer’s sire, and calf sex (when *P *< 0.25) as fixed effects. Metabolites and uterine blood flow included day and nutritional plane × day as repeated measures. Circulating glucose was less (*P* = 0.05) for NR dams than CON. Circulating urea N and triglycerides were less (*P* ≤ 0.05), but non-esterified fatty acids were greater (*P* ≤ 0.05), for NR at most timepoints after treatment initiation. Post-calving, NR dams were 62.1 kg and 2.1 BCS less (*P* < 0.01) than CON. Moreover, NR had less (*P* < 0.01) backfat and longissimus muscle area, but similar (*P* = 0.72) shoulder height compared with CON. Heart rate was less (*P* < 0.01) for NR dams than CON after treatment initiation. Nutritional plane did not affect (*P *≥ 0.15) ipsilateral, contralateral, or total uterine artery blood flow. Number of cotyledons was greater (*P* = 0.03), average cotyledon weight was less (*P* = 0.04), and total placental weight tended to be less (*P* = 0.10) for NR than CON. Cotyledonary relative mRNA expression of GLUT1 was greater (*P* = 0.04) and SNAT2 tended to be greater (*P* = 0.07) for NR, but other nutrient transporters, angiogenic factors, and PAG2 were not affected (*P *≥ 0.13). Calves born to NR dams weighed 14.4% (*P* = 0.03) less at birth and had smaller (*P* ≤ 0.03) heart girth and volume than CON. Despite catabolizing maternal tissue stores, beef heifers experiencing late gestational nutrient restriction had altered circulating metabolites and smaller placentas, independent of a reduction in uterine blood flow, which compromised fetal growth.

## Teaser

First-parity beef females that were nutrient restricted during late gestation lost maternal body weight and mobilized adipose and muscle tissue stores. Nutrient restricted females had altered circulating metabolites and smaller placentas, independent of a reduction in uterine blood flow or major changes in cotyledonary nutrient transporter expression, which collectively led to decreased calf birth weight.

## Introduction

Maternal nutrient imbalances (greater requirements vs. intake) during pregnancy across livestock species can result in intrauterine growth restriction and/or developmentally-program postnatal productivity (Wu et al., 2006; Reynolds et al., 2010). Late gestation is a particularly vulnerable period for grazing beef females as they are often faced with limited nutrient availability due to reduced forage quantity or poor forage quality (DelCurto et al., 2000; Caton and Hess, 2010) as late gestational energy and protein requirements increase exponentially to support the growth of the uteroplacenta, fetus, and mammary gland (Ferrell et al., 1976; NASEM, 2016). Furthermore, first-parity beef females are still growing during pregnancy; thus, there is competition for nutrients to be partitioned among uteroplacental, fetal, mammary, and maternal tissue growth (Short and Adams, 1988).

In a subset of late gestational undernutrition studies, calf birth weight was decreased approximately half of the time (Corah et al., 1975; Bellows and Short, 1978; Kroker and Cummins, 1979; Kennedy et al., 2016a) but was not affected the other half of the time (Anthony et al., 1986; Hough et al., 1990; Martin et al., 1997). Although these studies were conducted by different laboratories and varied in the severity, duration, and methods by which nutrient restriction was applied, they represent the 2 outcomes commonly observed. Fetal growth is dictated by uteroplacental nutrient transport capacity and includes factors such as available nutrients in maternal circulation, blood flow to the gravid uteroplacenta, placental size, abundance of nutrient transporters, and placental morphology (Fowden et al., 2006), but these were rarely determined in the aforementioned studies. Our lab previously observed that beef heifers fed 70% of energy and protein requirements during late gestation prioritized partitioning nutrients to fetal growth over maternal growth, catabolizing maternal tissue stores and maintaining total uterine artery blood flow and placental mass, while also increasing transcription of some cotyledonary nutrient transporters, which together allowed calf birth weight to be spared (Redifer et al., 2023, 2024). While this previous study demonstrated that pregnant beef heifers can be resilient when faced with late gestational undernutrition, it is important to elucidate the uteroplacental mechanisms that are compromised when the classically expected negative outcome occurs and intrauterine growth is restricted. It is rare in the literature for the same research model to be conducted in successive experiments and report divergent outcomes, especially with this breadth of variable measured.

The current experiment investigated the impacts of late gestational nutrient restriction in individually-fed first-parity beef females on prenatal nutrient availability to the offspring. We hypothesized that late gestational nutrient restriction alters nutrient concentrations in maternal circulation, causing mobilization of maternal adipose and muscle reserves to meet nutrient demands. Additionally, we hypothesized that late gestational nutrient restriction lessens blood flow to the uterus, decreases placental growth, and reduces cotyledonary nutrient transporter and angiogenic factor abundance, ultimately decreasing uteroplacental nutrient transport capacity and negatively affecting fetal growth.

## Materials and Methods

The University of Missouri Animal Care and Use Committee approved animal care and use in this study (Protocol #9877), which took place at the University of Missouri Beef Research and Teaching Farm (Columbia, MO, USA).

### Animal management and diets

Fall-calving Simmental-Angus crossbred beef heifers born to a single cowherd and developed at the University of Missouri Beef Research and Teaching Farm (*n* = 47) that were either sired by a Simmental-Angus sire (AI) or Angus sires (natural service) were bred for use in this study. These females were split into 2 similarly-sized groups for synchronization of ovulation using the 7-d CO-Synch + controlled internal drug release (**CIDR**) protocol. Following CIDR-removal on either December 16, 2020, or December 26, 2020, heifers [458 ± 12 d (SD throughout methods) of age at breeding] were bred to a single Simmental-Angus sire via a split-time artificial insemination (**AI**) protocol. Heifers were fitted with heat detection patches and monitored for natural return to estrus approximately 3 wk after initial breeding and were artificially inseminated to the same sire to obtain sufficient numbers. This resulted in 4 breeding groups occurring approximately every 10 to 11 d. Via transrectal ultrasonography, pregnancy was confirmed approximately 35 d after AI for each group, and fetal sex was determined between days 60 and 80 of gestation. From AI until animals were moved to the individual feeding facility, heifers were managed as a group in 18 × 61 m drylot pens with ad libitum access to tall fescue-based hay. During this time, heifers were supplemented with whole corn and dried distillers grains with solubles (**DDGS)** to meet or exceed nutrient requirements and maintain a target body condition score (**BCS**; 1 to 9 scale, 1 = emaciated, 9 = obese; Wagner et al., 1988) of 5 to 6.

From the pregnant females, 26 heifers [initial body weight (**BW**) = 472 ± 33 kg, initial BCS = 5.4 ± 0.5] were allocated by BW, BCS, heifer’s sire (AI vs. natural service), fetal sex, and expected calving date to 1 of 2 late gestational nutritional planes from day 160 of gestation to parturition. Control (**CON**; *n* = 13) heifers were individually-fed 100% of metabolizable energy (**ME**) and metabolizable protein (**MP**) requirements for maintenance, pregnancy, and growth, whereas nutrient restricted (**NR**; *n* = 13) heifers were individually-fed 70% of ME and MP requirements. Both the CON and NR groups consisted of 6 dams carrying male offspring and 7 dams carrying female offspring, with all offspring sired by a single SimAngus sire.

Animal management and nutritional plane treatments applied during late gestation were similar to those described in Redifer et al. (2023). By breeding group, on day 143 ± 0.8 of gestation, pregnant heifers were moved to partially-covered pens (3.7 × 15.8 m) equipped with a Calan gate feeding system (Calan Broadbent Feeding System, American Calan, Northwood, NH, USA) for acclimation to individual feeding in the electronic gate system. Heifers were allocated to 1 of 11 pens and penned by nutritional plane (*n* = 2 to 3 heifers/pen).

Late gestational ME and MP requirements were estimated using an expected calf birth weight of 34 kg, a projected maternal average daily gain of 0.36 kg/d, and the equations provided in Redifer et al. (2023). Metabolizable energy for maintenance was based on data for heifers in confinement (0.138 Mcal ME/kg non-gravid BW^0.75^; Freetly and Hales, personal communication). The equation used for ME for conceptus was published by Freetly et al. (2005). Equations from NASEM (2016) were utilized for ME for gain and MP for maintenance, conceptus, and gain. Metabolizable protein requirements were converted to crude protein (**CP**) requirements using the equation CP (g/d) = MP / 0.64, based on 80% true protein × 80% digestibility (NASEM, 2016). Nutrient requirements were adjusted weekly for the current day of gestation to account for exponentially increasing nutrient needs of advancing pregnancy, and BW were updated every 21 d. Requirements were then multiplied by a factor of 1.0 or 0.7 to obtain final ME and CP targets for each individual CON and NR heifer, respectively. Control heifers were expected to maintain a BCS of 5 to 6 and gain maternal (non-gravid) BW, reaching 80% of their estimated mature BW (490 kg for mature BW of 613 kg) by parturition.

Nutritional planes were initiated between days 158 and 160 of gestation (after completion of all initial data collection for all animals within a breeding group) by feeding animals individually in the Calan gate system. Diets were based on poor-quality chopped sorghum sudan hay [*Sorghum* × *drummondii*; 1.70 Mcal ME/kg, 6.46% CP, 72.7% neutral detergent fiber, 51.1% acid detergent fiber; dry matter (**DM**) basis] that had been stored under roof and from the same lot as that used in Redifer et al. (2023). Feeding poor-quality forage allowed animals fed both nutritional planes to consume ad libitum hay, preventing any pica in the NR dams without exceeding the ME and CP targets at any point. Sorghum sudan hay was initially offered at 1.56% BW on DM basis and then adjusted based on refusals. Adequate hay refusals were always present for all females to indicate ad libitum feeding and were weighed before removing twice weekly to determine intake.

Based on expected individual hay intakes, heifers were supplemented daily with whole corn (3.18 Mcal ME/kg, 9.28% CP; DM basis), DDGS (3.22 Mcal ME/kg, 32.9% CP; DM basis), and soyhull pellets (2.89 Mcal ME/kg, 11.4% CP; DM basis) to meet their assigned nutritional plane. Supplement for each heifer was reformulated weekly to meet the increasing ME and CP targets of advancing gestation, and then supplement for each day was weighed for each animal individually. Hay was offered ad libitum, and the NR nutritional plane was achieved solely by a reduction in supplement provided, resulting in a decrease in ME and MP intake. The corn:soyhull and DDGS:soyhull ratios remained similar for animals within a nutritional plane but varied between nutritional planes as necessary to meet each heifer’s ME and CP targets.

Supplement was delivered in a feed pan placed on top of previously-delivered hay in the Calan gate bunks to prevent wastage. Supplement was fed every morning at approximately 0700 h and was consumed before morning delivery of hay. In the few instances when supplement was not completely consumed by a female, the refusals were removed and added to the normal allotment of supplement on the following day. Hay was delivered into the Calan gate bunks in 2 equal portions to each individual heifer every morning (0730 h) and evening (1900 h). Heifers had ad libitum access to water. Loose mineral and vitamin supplement was included in each individual’s supplement at 60 g/d (16.0% Ca min., 19.2% Ca max., 12.0% P min., 13.0% NaCl min., 15.6% NaCl max., 5.0% Na min., 6.0% Na max., 0.50% Mg min., 1,420 mg/kg Cu min., 37.6 mg/kg Se min., 7,123 mg/kg Zn min., 4,684 mg/kg Mn min., 141 mg/kg I min., 352,440 IU/kg Vitamin A min., 88,123 IU/kg Vitamin D min., 881 IU/kg Vitamin E min.; Breeder 12 Mineral, MFA Inc., Columbia, MO, USA), which allowed intake to meet or exceed the most-limiting micronutrient (Vitamin A). Approximately once monthly, pen floors were scraped clean and rebedded with fresh sawdust.

Nutrient analysis of the hay and supplement feedstuffs was conducted throughout the experiment to reformulate the daily supplement to meet ME and CP targets as described in Redifer et al. (2023). Hay was sampled prior to grinding each group of 6 bales, and whole corn, DDGS, and soyhull pellets were subsampled at the start of each load. Representative subsamples of the chopped hay, supplement feedstuffs, and hay refusals were also collected and analyzed after the experiment to calculate nutrient intakes during individual feeding as reported in Redifer et al. (2023). Dry matter was determined for weekly hay composite samples and weekly orts subsamples (for each individual). Nutrient analysis was conducted for monthly composited samples of whole corn, DDGS, and soyhull pellets, and nutrient analysis of 6-bale grinding groups was used for hay. Dry matter, ME, and CP intakes from each ingredient were calculated and summed by day, then averaged by week. Intakes were calculated through the last complete 3- or 4-d period of hay delivery prior to day of calving; thus, nutrient intakes for the week beginning on day 265 of gestation were not included for 1 CON and 2 NR heifers that calved from days 262 to 266 of gestation.

It was observed that maternal performance for both nutritional planes was not meeting expectations and markedly less than the previous study (Redifer et al., 2023) at approximately day 223 of gestation. Thereafter, supplement delivery was formulated to provide 105% of targeted ME and CP targets for each nutritional plane, which resulted in ME intake for both nutritional planes being greater than estimated ME requirements until calving. Crude protein should have followed a similar pattern, but the daily composite samples for whole corn and soyhulls pellets fed during approximately the last month of gestation had lower CP concentrations than the initial samples used for supplement formulation, and CP intake unexpectedly fell below estimated requirements.

### Maternal gestational performance

Jugular blood samples and consecutive 2-d BW were collected before nutritional plane treatment allocation (days 158 and 159 of gestation). After treatment initiation, gestational BW and jugular blood samples were collected at 21-d intervals occurring on days 181, 202, 223, 244, and 265 of gestation (2-d BW every 42 d and on day 265 of gestation). These occurred immediately before the morning delivery of supplement and hay and within ± 2 d of the actual day of gestation. Dam BCS was assessed by the same 2 trained technicians on days 158, 202, 244, and 265 of gestation, and scores were averaged. Backfat thickness and longissimus muscle area between the 12th and 13th ribs was measured by a trained technician on days 158, 202, and 244 of gestation using an Aloka 500-SSV ultrasound machine (Aloka Co. Ltd, Tokyo, Japan) with an Aloka 17 cm 3.5 MHz linear transducer (UST-5044-3.5) and standoff. Backfat thickness was measured on 2 separate images chute-side and was determined at a point three-quarters the length of the longissimus muscle from the spine. Both images were saved onto an external memory source, and the longissimus muscle cross-section was traced using ImageJ software on a computer. Each image was traced twice by the same 2 technicians, resulting in 4 measurements averaged for analysis. On days 158 and 159 of gestation, shoulder height was determined utilizing a frame size measuring stick (Sullivan Supply Inc., Dunlap, IA, USA) and the measurements averaged.

### Uterine artery blood flow

Uterine blood flow was determined before nutritional plane treatment allocation (day 157 of gestation) and at 21-d intervals occurring on days 181, 202, 223, and 244 of gestation (collected within ± 2 d of the actual day of gestation). Hemodynamics of the ipsilateral and contralateral uterine arteries were assessed via transrectal color Doppler ultrasonography using an Aloka Prosound α6 (Hitachi Aloka, Tokyo, Japan) equipped with a 7.5 MHz convex finger transducer (Aloka USD-995) as described in Redifer et al. (2024).

On each ultrasound day, 3 or 4 ultrasound scans (each scan including ≥ 3 cardiac cycle waveforms) were obtained for the ipsilateral and contralateral uterine arteries, with all measurements made and data recorded chute-side. All ultrasound examinations were conducted between 0745 and 1245 h. Ultrasound examinations lasted approximately 40 min for each female, but over half of the time was used for a technician capturing measurements on the ultrasound machine and recording data, during which transrectal examination did not occur. For all uterine blood flow data collection, a single trained technician conducted the scans, and a separate single trained technician captured the measurements on the ultrasound machine. All uterine blood flow variables are reported for the ipsilateral and contralateral uterine arteries separately, except maternal heart rate which was averaged for the 2 sides. Total uterine blood flow for each female was calculated as the sum of the blood flow of the ipsilateral and contralateral uterine arteries.

Flow gain was set between 40 and 45 (average: 41.1) for the ipsilateral side and between 40 and 50 (average: 43.1) for the contralateral side. The average angle of insonation for obtaining blood flow waveforms was maintained between 63° and 79° (average: 71.4°) for the ipsilateral uterine artery and between 60° and 76° (average: 67.9°) for the contralateral uterine artery. The average angle of insonation from previous scans was used to maintain consistency of angle within each side for a single female across all 5 timepoints. To determine if angle of insonation affected blood flow data interpretation, PROC MIXED (SAS 9.4, SAS Institute Inc., Cary, NC) was used to determine the effects of nutritional plane, day, and nutritional plane × day (repeated measures as described below for uterine blood flow date, but no covariates in the model). Ipsilateral average angle of insonation tended to be affected (*P* = 0.06) by the nutritional plane × day of gestation interaction, but interactive means were not affected (*P* ≥ 0.49) by nutritional plane on any given day of gestation, and all angle means for both nutritional planes were between 71.0 and 71.8 at each day. Contralateral average angle of insonation was not affected (*P* ≥ 0.36) by the late gestational nutritional plane × day of gestation interaction or the main effect of nutritional plane.

### Calving data and sample collection

During the peripartum period, indoor and outdoor lighting allowed for continuous monitoring of females. Heifers were closely monitored 24 h per day by trained personnel to detect when heifers were in stage II of parturition (Noakes et al., 2001). Once stage II was detected, the heifer was continuously monitored. Calving assistance was provided if there was a prolonged duration since first appearance of fetal membranes or feet (>1 h without progress) or if progress slowed during contractions based on expertise of trained personnel. The average calving date was September 24 ± 6.5 d (range September 12 to October 9). Calves were removed from their dams immediately post-calving for further research objectives not included in this paper.

### Expelled placental collection and processing

Dams were continuously monitored post-calving until placentas were collected after natural expulsion and processed as described in Redifer et al. (2024). Within 10 min of collection, tissue from 2 representative ipsilateral cotyledons was excised, pooled, flash frozen on dry ice, and stored at −80°C until ribonucleic acid (**RNA**) extraction. Placentas were rinsed, separated into ipsilateral and contralateral sides, refrigerated, dissected, and stored at −20°C until all dams had calved. Dry matter weights were determined for the 4 sections separately (ipsilateral cotyledonary, ipsilateral intercotyledonary, contralateral cotyledonary, contralateral intercotyledonary).

### Calf size at birth

Prior to provision of colostrum, calf sex was recorded and birth BW was determined (1.0 ± 0.3 h of age) using a digital walk-on platform scale. Calf shoulder to rump length, heart girth, abdominal girth, flank girth, cannon circumference, cannon length, coronet circumference, and shoulder height were measured, and ponderal index, heart girth:length, and volume were calculated as described in Redifer et al. (2023). Additionally, calf longissimus muscle area was measured via carcass ultrasound on day 2 of age as described in Redifer et al. (2023).

### Maternal performance post-calving

Post-calving, hay and supplement provision was not changed from the most recent gestational allocation until all post-calving data collection was complete for a female. A post-calving jugular blood sample was obtained from each dam at 0.63 ± 0.35 h post-calving, and post-calving 2-d BW were collected on days 1 and 2 post-calving between 0330 and 0900 h. In conjunction with BW, maternal backfat thickness and longissimus muscle area were collected on day 1 post-calving, and maternal shoulder height was collected on days 1 and 2 post-calving, as described for gestational timepoints. During the first week postpartum, maternal BCS (2.4 ± 1.4 d post-calving) was collected as described for gestational timepoints. Average daily gain (gravid), maternal average daily gain (non-gravid), BCS change, backfat thickness change, longissimus muscle area change, and shoulder height change from treatment initiation to pre-calving (gravid average daily gain only) and post-calving were calculated. Maternal average daily gain was determined by subtracting the non-gravid BW at treatment initiation from the post-calving BW. The gravid uterine weight at treatment initiation was calculated using the equation described above from NASEM (2016) and the known calf birth BW of a respective dam, which was used to calculate non-gravid BW.

### Circulating metabolite analyses

Maternal blood samples were collected, plasma and serum were harvested, and plasma glucose, serum urea N, plasma triglycerides, and serum non-esterified fatty acids (**NEFA**) were analyzed as described in Redifer et al. (2023). For each assay, samples were analyzed in duplicate, and pooled control samples were used. The intraassay and interassay CV were 3.4% and 2.6% for plasma glucose, 3.2% and 3.5% for serum urea N, 2.7% and 1.7% for plasma triglycerides, and 2.2% and 3.1% for serum NEFA, respectively.

### Cotyledonary relative mRNA expression

Cotyledonary tissue was pulverized, total RNA was extracted, and complimentary deoxyribonucleic acid was synthesized as described in Redifer et al. (2024). Ribonucleic acid quantity (average: 174 ng/µL, range: 70 to 311 ng/µL) and quality (average: 6.5 RNA integrity number, range: 5.2 to 8.1 RNA integrity number) were assessed. Bovine primer sequences obtained from Bio-Rad Laboratories (PrimePCR SYBR Green Assay) were selected for genes involved in placental glucose and fructose transport [GLUT1 (SLC2A1), GLUT3 (SLC2A3), GLUT4 (SLC2A4), and GLUT5 (SLC2A5)], amino acid transport [4F2hc (SLC3A2), CAT1 (SLC7A1), LAT1 (SLC7A5), LAT2 (SLC7A8), and SNAT2 (SLC38A2)], angiogenesis [VEGFA (vascular endothelial growth factor A), NOS3 (nitric oxide synthase 3), FLT1 (VEGFA receptor 1), KDR (VEGFA receptor 2), and GUCY1B3 (nitric oxide receptor)], PAG2 (pregnancy-associated glycoprotein 2), and G3PDH (glyceraldehyde 3-phosphate dehydrogenase). Primer efficiencies were 97% to 110%.

Analysis of messenger RNA (**mRNA**) expression was completed by quantitative real-time polymerase chain reaction as described in Redifer et al. (2024). Each 96-well plate contained a pooled internal control and a no template control, with all samples analyzed in duplicate. Target mRNA cycle threshold values for each gene were normalized to a reference gene (G3PDH) with mRNA expression calculated relative to the pooled internal control using the 2^−ΔΔCT^ method (Livak and Schmittgen, 2001). Cycle threshold values for G3PDH were not affected (*P* = 0.98; 22.8 vs. 22.8 ± 0.3) by late gestational nutritional plane. For mRNA expression data, if a 2^−ΔΔCT^ was > 3 SD away from the group mean for a particular gene, it was considered an outlier (*n* = 2 for NR) and removed from the dataset. The intraassay and interassay CV for the pooled internal controls were 0.4% and 1.0%, respectively.

### Statistical analyses

Four heifers (3 CON and 1 NR) were removed from the study completely due to stillbirths, positive fetal Neospora cases, or unviable offspring, resulting in CON *n* = 10 and NR *n* = 12. For the 3 heifers (1 CON and 2 NR) that calved around the day 265 of gestation, data collection at that timepoint was incomplete. Longissimus muscle area on day 202 of gestation was not determined for 1 CON and 1 NR heifer. Ipsilateral cotyledonary tissue was not frozen for relative mRNA expression for 1 NR heifer. The RNA integrity number was < 5 for 1 CON and 1 NR heifer; thus, they were not included in cotyledonary mRNA expression analyses. For 3 heifers (1 CON and 2 NR), placentas were collected and cotyledonary tissue was frozen but deemed incomplete upon inspection, and not dissected for placental size. For 1 CON heifer, the ipsilateral intercotyledonary tissue remained heavily contaminated with sawdust after rinsing, and the DM was not reported.

Dry matter and nutrient intakes (by day), maternal BW and body composition (by day), placental size, cotyledonary mRNA expression, and calf size at birth were analyzed using the MIXED procedure in SAS 9.4 (SAS Institute Inc., Cary, NC) with late gestational nutritional plane as a fixed effect and animal as the experimental unit. Gestational circulating metabolites and uterine blood flow included late gestational nutritional plane, day of gestation, and their interaction as fixed effects. These were considered repeated measures using animal as the subject and the majority best-fit covariance structure (based on Akaike Information Criterion, Bayesian Information Criterion, and corrected Bayesian Information Criterion) specific for each variable (chosen from unstructured, compound symmetry, heterogeneous compound symmetry, autoregressive, and heterogeneous autoregressive). Circulating metabolites at 1 h post-calving were analyzed separately with late gestational nutritional plane as a fixed effect. For all measures except DM and nutrient intakes, Julian date of treatment initiation (to remove variation in breeding date and age of dam), heifer’s sire (AI or natural service), and calf sex (if *P* < 0.25) were included as covariates (fixed effects). For cotyledonary relative mRNA expression, time from parturition to cotyledonary tissue sampling was also included as a covariate. PROC TTEST was used to test if average daily gain and changes in BCS, backfat thickness, longissimus muscle area, and shoulder height were different than 0 within nutritional planes. The proportion of births that required assistance at calving or were abnormal presentations were evaluated by the 2-sided Fisher’s exact test using the FREQ procedure of SAS. Means were separated using least significant difference and considered different when *P* ≤ 0.05 and tendencies were considered when 0.05 < *P* ≤ 0.10. In the absence of interactions, main effects are discussed.

## Results

### Nutrient intake during gestation

**Figure 1.**
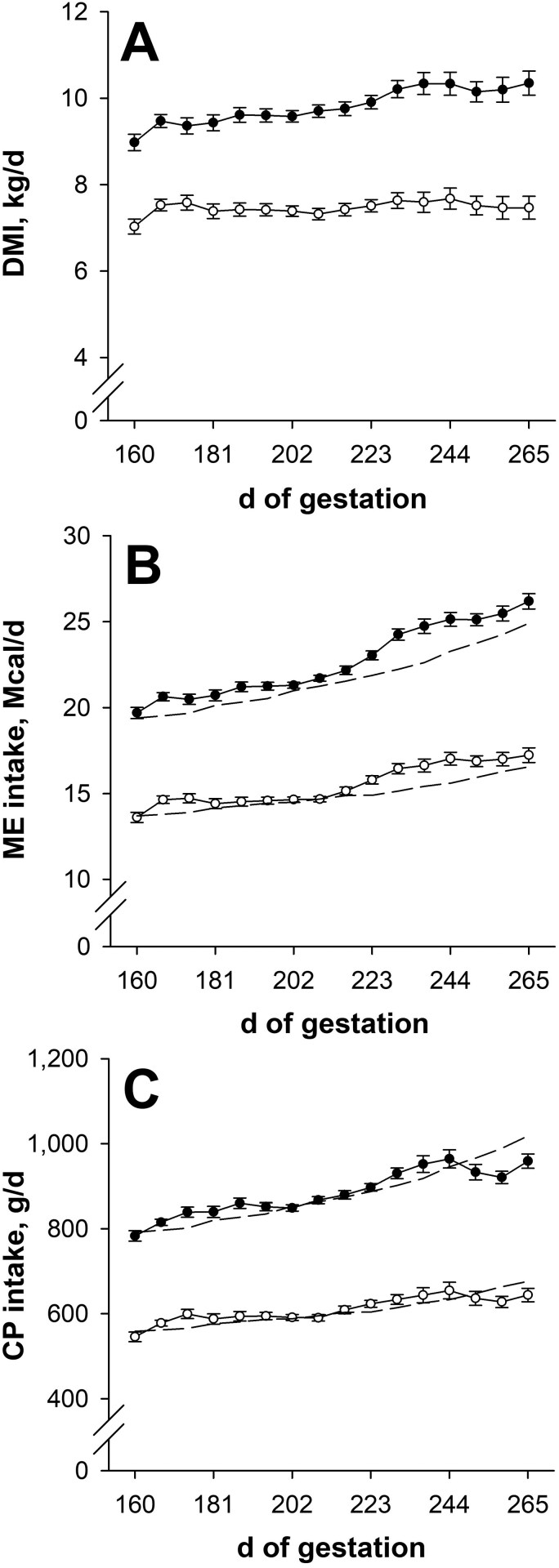
Dry matter intake (DMI; panel A), metabolizable energy intake (ME; panel B), and crude protein intake (CP; panel C) from day 160 of gestation to parturition. Solid circles (●) represent primiparous beef females individually-fed 100% (Control; *n* = 9 or 10) and open circles (○) represent primiparous beef females individually-fed 70% (Nutrient restricted; *n* = 10 to 12) of metabolizable energy and metabolizable protein requirements for maintenance, pregnancy, and growth from day 160 of gestation to parturition. Least squares means ± SEM are presented. Nutritional plane means differ (*P* ≤ 0.001) on all days for each measure. Targeted weekly energy and protein intakes are represented by the dashed lines.

Dry matter intake, ME intake, and CP intake were less (*P* < 0.001; Fig. 1A to 1C) for NR dams compared with CON during late gestation. For CON dams, weekly ME and CP intakes during late gestation averaged 104.7% (weekly range: 101.4% to 109.3%) and 100.6% (weekly range: 93.1% to 104.7%) of their ME and CP targets, respectively. Weekly ME and CP intakes of NR dams averaged 104.0% (weekly range: 99.4% to 109.1%) of their ME targets and 100.9% (weekly range: 94.6% to 106.0%) of their CP targets during late gestation. Weekly dry matter intake (**DMI**) during late gestation averaged 23.9% less (*P* < 0.001; range: 19.0% to 27.9%) for NR dams compared with CON. Hay DMI was not affected (*P* ≥ 0.27; data not shown) by late gestational nutritional plane.

### Maternal gestational performance and circulating metabolites

**Figure 2.**
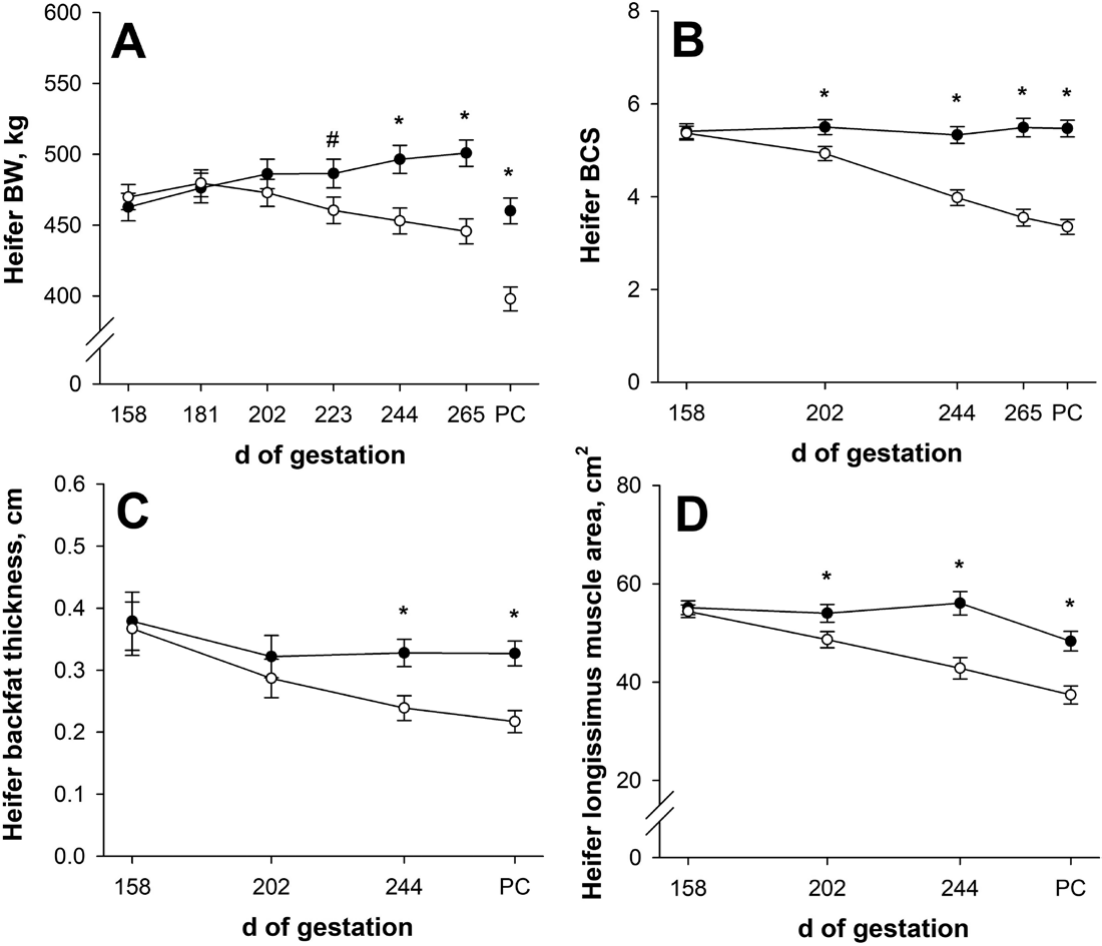
Effects of late gestational nutritional plane on heifer body weight (BW; panel A), body condition score (BCS; panel B), backfat thickness (panel C), and longissimus muscle area (panel D) from day 158 of gestation until day 1 post-calving (PC). Solid circles (●) represent primiparous beef females individually-fed 100% (Control; *n* = 9 or 10) and open circles (○) represent primiparous beef females individually-fed 70% (Nutrient Restricted; *n* = 11 or 12) of ME and MP requirements for maintenance, pregnancy, and growth from day 160 of gestation to parturition. Dam BCS was assessed on a 1 to 9 scale (1 = emaciated, 9 = obese). Least squares means ± SEM are presented. *Nutritional plane means differ (*P* ≤ 0.05). ^#^Nutritional plane means tend to differ (0.05 < *P* ≤ 0.10).

Initial BW, BCS, backfat thickness, longissimus muscle area, and shoulder height did not differ (*P* ≥ 0.60; Table 1; Fig. 2A to 2D) between late gestational nutritional planes. On day 223 of gestation, pregnant heifer BW tended to be less (*P* = 0.08; Fig. 2A) for NR dams than CON. Pregnant heifer BW was less (*P* ≤ 0.005) for NR dams for the remainder of gestation, where NR dams weighed 11.0% less (*P* < 0.001; 446 vs. 501 ± 9 kg) than CON on day 265 of gestation. Pregnant heifer average daily gain (gravid weight) from treatment initiation until day 265 of gestation was affected (*P* < 0.001; Table 1) by nutritional plane, where CON dams gained (*P* < 0.001) pregnant BW while NR dams decreased (*P* < 0.001) pregnant BW. Maternal post-calving BW was 13.5% less (*P* < 0.001; 398 vs. 460 ± 9 kg) for NR dams compared with CON. Maternal average daily gain (non-gravid) from day 158 of gestation until post-calving was less (*P* < 0.001; Table 1) for NR dams compared with CON. For NR dams, maternal BW during late gestation decreased (*P* < 0.001), but for CON dams, maternal average daily gain was not different (*P* = 0.22) than 0.

**Table 1.** Effects of late gestational nutritional plane of primiparous beef females on maternal growth and body composition changes during gestation

	Nutritional plane^1^			
Item	CON	NR	SEM^2^	*P*-value
Shoulder height, cm				
Day 158 of gestation	119	119	1	0.96
Post-calving	120	121	2	0.72
Average daily gain (gravid), kg/d				
Days 158 to 265 of gestation	0.37	−0.23	0.03	<0.001
Maternal average daily gain (non-gravid), kg/d				
Day 158 of gestation to post-calving^3^	0.05	−0.57	0.04	<0.001
Body condition score change^4^				
Day 158 of gestation to post-calving	0.07	−2.02	0.08	<0.001
Backfat thickness change, cm				
Day 158 of gestation to post-calving	−0.051	−0.150	0.043	0.11
Longissimus muscle area change, cm^2^				
Day 158 of gestation to post-calving	−6.82	−17.00	1.58	<0.001
Shoulder height change, cm				
Day 158 of gestation to post-calving	1.21	1.97	0.43	0.21

^1^Primiparous dams were individually-fed either 100% (Control; CON) or 70% (Nutrient Restricted; NR) of metabolizable energy and metabolizable protein requirements for maintenance, pregnancy, and growth from day 160 of gestation to parturition.

^2^Standard error of the mean for CON (*n* = 10) and NR (*n* = 11 or 12).

^3^Non-gravid body weight on day 158 of gestation determined by subtracting the gravid uterine weight.

^4^1 to 9 scale (1 = emaciated, 9 = obese).

On day 202 of gestation, NR dams had less (*P* = 0.02; Fig. 2B) BCS than CON, and BCS differences remained (*P* < 0.001) throughout gestation, where NR dams had 35.3% less (*P* < 0.001; 3.55 vs. 5.49 ± 0.20) BCS on day 265 of gestation compared with CON. On day 202 of gestation, maternal backfat thickness was not affected (*P* = 0.46; Fig. 2C) by nutritional plane, but by day 244 of gestation, maternal backfat thickness was 27.1% less (*P* = 0.008; 0.239 vs. 0.328 ± 0.022 cm) for NR dams compared with CON. Post-calving maternal BCS was 38.8% less (*P* < 0.001; 3.35 vs. 5.47 ± 0.18) and maternal backfat thickness was 33.6% less (*P* < 0.001; 0.217 vs. 0.327 ± 0.020 cm) for NR dams than CON. Body condition score change during late gestation was affected (*P* < 0.001; Table 1) by nutritional plane, but backfat thickness change was not affected (*P* = 0.11). However, CON dam BCS change and backfat thickness change were not different (*P* ≥ 0.15) than 0, while NR dams decreased (*P* ≤ 0.01) in BCS and backfat thickness from day 158 of gestation until post-calving.

On day 202 of gestation, NR dams had smaller (*P* = 0.04; Fig. 2D) longissimus muscle area than CON, and longissimus muscle area differences remained (*P* < 0.001) throughout gestation, where NR dams had 23.6% less (*P* < 0.001; 42.8 vs. 56.0 ± 2.4 cm^2^) longissimus muscle area on day 244 of gestation compared with CON. Post-calving maternal longissimus muscle area was 22.6% less (*P* < 0.001; 37.4 vs. 48.3 ± 2.0 cm^2^) for NR dams than CON, but post-calving maternal shoulder height was not affected by nutritional plane (*P* = 0.72; Table 1). From day 158 of gestation until post-calving, dams from both nutritional planes decreased (*P* ≤ 0.003) longissimus muscle area, but longissimus muscle area loss for NR dams was greater (*P* < 0.001; Table 1) than CON. Dams from both nutritional planes increased (*P* ≤ 0.02) shoulder height during late gestation, and shoulder height change was not affected (*P* = 0.21; Table 1) by nutritional plane.

**Figure 3.**
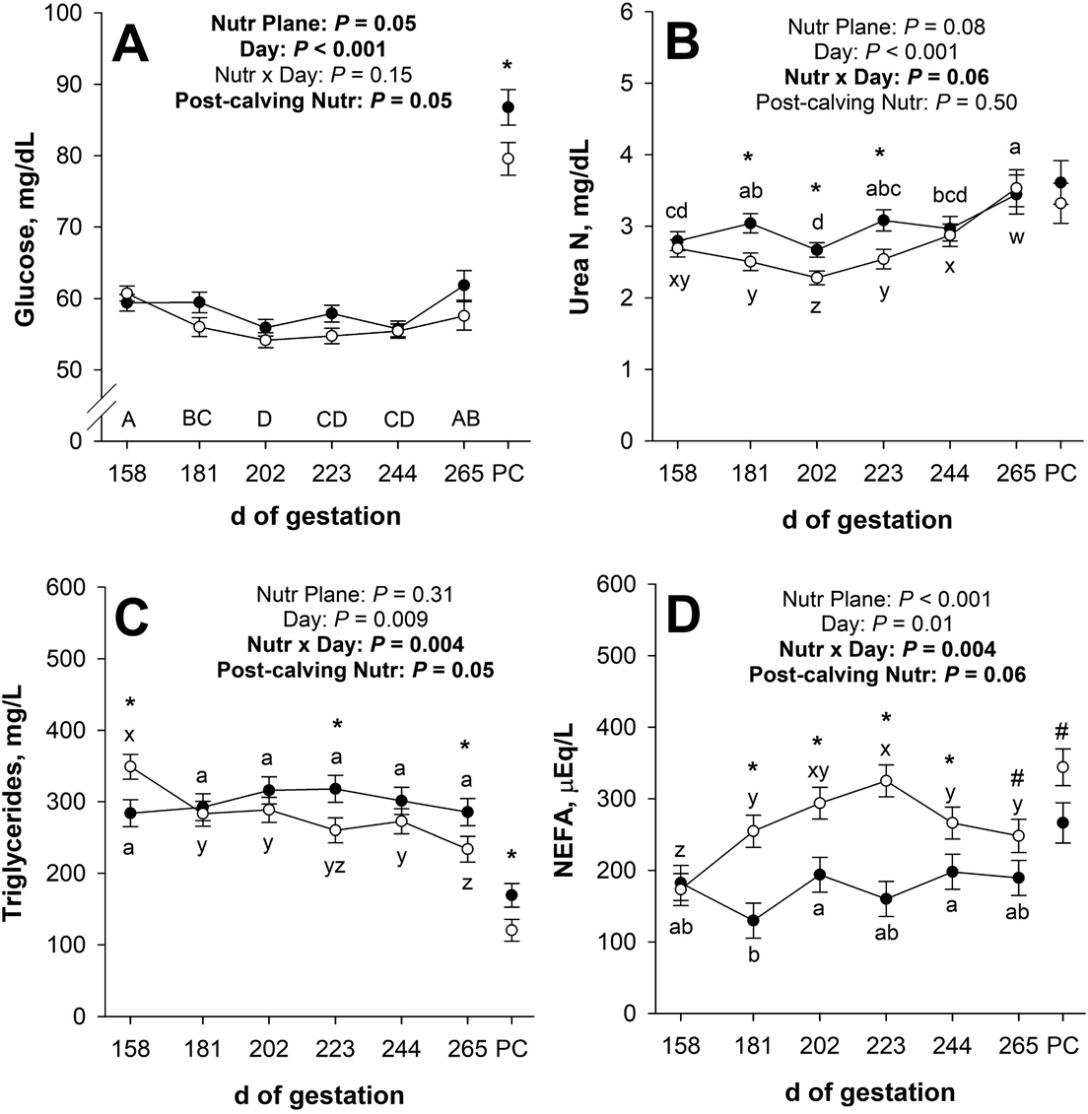
Effects of late gestational nutritional plane on maternal plasma glucose (panel A), serum urea N (panel B), plasma triglycerides (panel C), and serum non-esterified fatty acids (NEFA; panel D) from day 158 of gestation until parturition. Solid circles (●) represent primiparous beef females individually-fed 100% (Control; *n* = 10) and open circles (○) represent primiparous beef females individually-fed 70% (Nutrient Restricted; *n* = 11 or 12) of metabolizable energy and metabolizable protein requirements for maintenance, pregnancy, and growth from day 160 of gestation to parturition. Least squares means ± SEM are presented. Gestational circulating metabolites were considered repeated measures, and 1 h post-calving (PC) circulating metabolites were analyzed separately. *Nutritional plane means within day differ (*P* ≤ 0.05). ^#^Nutritional plane means within day tend to differ (0.05 < *P* ≤ 0.10). ^a,b,c,d^Means differ (*P* ≤ 0.05) for Control across days. ^w,x,y,z^Means differ (*P* ≤ 0.05) for Nutrient Restricted across days. ^A,B,C,D^Means for main effect of day differ (*P* ≤ 0.05).

Maternal plasma glucose concentrations during late gestation were not affected (*P* = 0.15; Fig. 3A) by the nutritional plane × day interaction. Nutrient restricted dams had less (main effect; *P* = 0.05) plasma glucose than CON. Additionally, there was an effect (*P* < 0.001) of day of gestation, where plasma glucose decreased (*P* ≤ 0.04) from days 158 to 202 of gestation and increased (*P* = 0.007) from days 244 to 265 of gestation. Plasma glucose at 1 h post-calving was less (*P* = 0.05) for NR dams compared with CON.

There tended to be a nutritional plane × day interaction (*P* = 0.06; Fig. 3B) for maternal serum urea N concentrations during late gestation. Initial serum urea N did not differ (*P* = 0.56) between nutritional planes. Serum urea N was less (*P* ≤ 0.008) for NR dams compared with CON on days 181, 202, and 223 of gestation, but serum urea N was not affected (*P* ≥ 0.69) by nutritional plane on days 244 or 265 of gestation. For CON dams, serum urea N increased (*P* = 0.05) from days 158 to 181 of gestation, decreased (*P* = 0.002) from days 181 to 202, increased (*P* = 0.002) from days 202 to 223, and increased (*P* = 0.04) again from days 244 to 265 of gestation. For NR dams, serum urea N decreased (*P* = 0.04) from days 181 to 202 and increased (*P* ≤ 0.03) from days 202 to 265 of gestation. At 1 h post-calving, serum urea N was not affected (*P* = 0.50) by nutritional plane.

Maternal plasma triglyceride concentration was affected (*P* = 0.004; Fig. 3C) by the nutritional plane × day interaction during late gestation. Initial plasma triglycerides were greater (*P* = 0.01) for NR dams compared with CON. Plasma triglycerides were not affected (*P* ≥ 0.27) by nutritional plane on days 181, 202, or 244 of gestation, but were less (*P* ≤ 0.05) for NR dams on days 223 and 265 of gestation compared with CON. For CON dams, plasma triglycerides did not change (*P* ≥ 0.18) throughout gestation; however, for NR dams, plasma triglycerides decreased (*P* ≤ 0.02) from days 158 to 181 and from days 244 to 265 of gestation and tended to decrease (*P* = 0.08) from days 202 to 223 of gestation. Plasma triglycerides were less (*P* = 0.05) for NR dams compared with CON at 1 h post-calving.

There was a nutritional plane × day interaction (*P* = 0.004; Fig. 3D) for maternal serum NEFA concentrations during late gestation. Initial serum NEFA did not differ (*P* = 0.78) between nutritional planes. Serum NEFA were greater (*P* ≤ 0.04) for NR dams than CON on days 181, 202, 223, and 244 of gestation and tended to be greater (*P* = 0.09) on day 265 of gestation. For CON dams, serum NEFA did not change (*P* ≥ 0.11) from days 158 to 181 or from days 202 to 265 of gestation, but serum NEFA increased (*P* = 0.05) from days 181 to 202 of gestation. For NR dams, serum NEFA increased (*P* = 0.007) from days 158 to d181 of gestation and was greater (*P* = 0.02) on day 223 compared with day 181, but serum NEFA decreased (*P* = 0.05) from days 223 to 244 of gestation. Serum NEFA at 1 h post-calving tended to be greater (*P* = 0.06) for NR dams compared with CON.

### Uterine artery blood flow

There was a late gestational nutritional plane × day interaction (*P* < 0.001; Table 2) for maternal heart rate during late gestation. Initial maternal heart rate did not differ (*P* = 0.57) between nutritional planes, but maternal heart rate was less (*P* ≤ 0.009) for NR dams than CON on days 181, 202, 223 and 244 of gestation. For CON dams, maternal heart rate increased (*P* ≤ 0.05) from days 157 to 181 and from days 202 to 223 of gestation. For NR dams, maternal heart rate decreased (*P* = 0.004) from days 157 to 181 of gestation, tended to decrease (*P* = 0.06) from days 181 to 202, but increased (*P* = 0.01) from days 202 to 223 of gestation.

**Table 2.** Effects of late gestational nutritional plane and day of gestation of primiparous beef females on uterine artery blood flow

	Nutritional plane^1^			Day of gestation						*P*-value^2^		
Item	CON	NR	SEM^3^	157	181	202	223	244	SEM^3^	Nutr	Day	Nutr × Day
Maternal heart rate, beats/min	–	–	–	–	–	–	–	–	–	0.009	<0.001	<0.001
CON	–	–	–	76.7^c^	81.9^ab^	80.9^bc^	85.2^a^	85.9^a^	2.4			
NR	–	–	–	78.6^w^	72.7^yz^^*^	68.8^z^^*^	73.9^xy^^*^	77.1^wx^^*^	2.2			
Ipsilateral uterine artery												
Peak systolic velocity, cm/s	–	–	–	–	–	–	–	–	–	0.27	<0.001	0.05
CON	–	–	–	269^b^	305^a^	306^ab^	325^a^	297^a^	34			
NR	–	–	–	281^z^	326^y^	359^xy^	365^x^	364^x^^#^	31			
End diastolic velocity, cm/s	144	168	10	133^c^	156^b^	167^a^	167^a^	158^ab^	9	0.11	<0.001	0.60
Mean velocity, cm/s	–	–	–	–	–	–	–	–	–	0.24	<0.001	0.10
CON	–	–	–	183^b^	219^a^	221^a^	236^a^	216^a^	23			
NR	–	–	–	199^z^	232^y^	258^x^	263^x^	259^x^^*^	21			
Pulsatility index	–	–	–	–	–	–	–	–	–	0.40	0.06	0.10
CON	–	–	–	0.785^a^	0.717^b^	0.686^b^	0.720^b^	0.708^b^	0.028			
NR	–	–	–	0.696^yz^^*^	0.694^yz^	0.675^z^	0.700^yz^	0.735^y^	0.025			
Resistance index	–	–	–	–	–	–	–	–	–	0.16	0.05	0.09
CON	–	–	–	0.537^a^	0.512^ab^	0.496^b^	0.519^ab^	0.513^ab^	0.013			
NR	–	–	–	0.489^z^^*^	0.490^z^	0.484^z^	0.506^yz^	0.520^y^	0.011			
Cross-sectional area, cm^2^	0.863	0.778	0.050	0.470^e^	0.671^d^	0.831^c^	0.989^b^	1.144^a^	0.053	0.23	<0.001	0.24
Blood flow, L/min	11.32	11.48	0.98	5.41^e^	9.09^d^	11.82^c^	14.53^b^	16.15^a^	1.02	0.91	<0.001	0.21
Blood flow, % of total	–	–	–	–	–	–	–	–	–	0.64	<0.001	0.07
CON	–	–	–	81.5^a^	81.1^a^	79.8^ab^	79.2^b^	77.1^c^	2.8			
NR	–	–	–	82.5^y^	82.3^y^	81.7^y^	79.9^z^	81.1^yz^	2.6			
Contralateral uterine artery												
Peak systolic velocity, cm/s	–	–	–	–	–	–	–	–	–	0.88	<0.001	0.04
CON	–	–	–	208^c^	221^bc^	233^b^	235^ab^	254^a^	16			
NR	–	–	–	187^z^	211^y^	237^x^	255^x^	246^x^	14			
End diastolic velocity, cm/s	95	94	6	76^c^	91^b^	103^a^	102^a^	101^a^	5	0.96	<0.001	0.63
Mean velocity, cm/s	150	147	10	120^d^	140^c^	156^b^	163^ab^	165^a^	7	0.82	<0.001	0.34
Pulsatility index	0.938	0.910	0.045	1.020^a^	0.920^b^	0.865^c^	0.890^bc^	0.926^bc^	0.037	0.66	<0.001	0.42
Resistance index	0.592	0.581	0.015	0.610^a^	0.580^bc^	0.562^c^	0.583^ab^	0.598^ab^	0.014	0.61	0.002	0.70
Cross-sectional area, cm^2^	0.292	0.279	0.033	0.151^e^	0.226^d^	0.279^c^	0.366^b^	0.406^a^	0.034	0.78	<0.001	0.88
Blood flow, L/min	2.83	2.52	0.36	1.10^e^	1.92^d^	2.68^c^	3.58^b^	4.12^a^	0.38	0.53	<0.001	0.15
Total blood flow, L/min	14.12	14.03	0.94	6.51^e^	11.01^d^	14.49^c^	18.11^b^	20.26^a^	1.01	0.94	<0.001	0.32
Total blood flow relative to calf birth weight^4^, mL·min^−1^·kg^−1^	539	647	54	274^e^	462^d^	616^c^	764^b^	847^a^	47	0.16	<0.001	0.32
Total blood flow relative to dam BW^5^, mL·min^−1^·kg^−1^	28.9	30.3	1.7	13.9^e^	22.9^d^	30.2^c^	38.3^b^	42.7^a^	1.8	0.54	<0.001	0.12

^1^Primiparous dams were individually-fed either 100% (Control; CON) or 70% (Nutrient Restricted; NR) of estimated metabolizable energy and metabolizable protein requirements for maintenance, pregnancy, and growth from day 160 of gestation to parturition.

^2^Probabilities of difference for late gestational nutritional plane (Nutr), day of gestation (Day), and their interaction.

^3^Standard error of the mean for CON (*n* = 10) and NR (*n* = 12).

^4^Calf birth weight determined at 1.0 ± 0.3 h (SD) of age (pre-suckling).

^5^Preprandial dam body weight determined at each respective timepoint.

^a,b,c,d,e^Means differ (*P* ≤ 0.05) for main effect of day (or for CON across days).

^w,x,y,z^Means differ (*P* ≤ 0.05) for NR across days.

^*^Within day, NR was different (*P* ≤ 0.05) than CON.

^#^Within day, NR tended to be different (0.05 ≤ *P* ≤ 0.10) than CON.

The nutritional plane × day of gestation interaction affected (*P* = 0.05; Table 2) ipsilateral uterine artery peak systolic velocity and tended to affect (*P* = 0.10) mean velocity. On day 244 of gestation, ipsilateral uterine artery mean velocity was greater (*P* = 0.05) and peak systolic velocity tended to be greater (*P* = 0.06) for NR dams compared with CON. The nutritional plane × day of gestation interaction tended to affect (*P* ≤ 0.10) ipsilateral uterine artery pulsatility and resistance indices, where ipsilateral uterine artery pulsatility and resistance indices were less (*P* ≤ 0.02) for NR dams compared with CON prior to nutritional plane initiation. Ipsilateral uterine artery end diastolic velocity, cross-sectional area, and blood flow were not affected (*P* ≥ 0.11) by the nutritional plane × day of gestation interaction or the main effect of nutritional plane, but all were affected (*P* < 0.001) by day of gestation. Ipsilateral uterine artery blood flow as a percentage of total blood flow tended to be affected (*P* = 0.07) by the nutritional plane × day of gestation interaction, but interactive means were not affected (*P* ≥ 0.30) by nutritional plane on any given day of gestation.

Contralateral uterine artery peak systolic velocity was affected (*P* = 0.04; Table 2) by the nutritional plane × day of gestation interaction, but interactive means were not affected (*P* ≥ 0.33) by nutritional plane on any given day of gestation. The nutritional plane × day of gestation interaction and the main effect of nutritional plane did not affect (*P* ≥ 0.15) any other contralateral uterine artery hemodynamics, but all variables were affected (*P* ≤ 0.002) by day of gestation.

The nutritional plane × day of gestation interaction and the main effect of nutritional plane did not affect (*P* ≥ 0.12; Table 2) total uterine artery blood flow, total blood flow relative to calf birth weight, and total blood flow relative to dam BW. Total uterine artery blood flow, total blood flow relative to calf birth weight, and total blood flow relative to dam BW increased (*P* ≤ 0.001) from days 157 to 244 of gestation.

### Placental size and cotyledonary relative mRNA expression

Ipsilateral dry cotyledonary, intercotyledonary, placental, and cotyledonary:intercotyledonary weights were not affected (*P* ≥ 0.12; Table 3) by late gestational nutritional plane. Ipsilateral number of cotyledons was greater (*P* = 0.003), but average dry cotyledon weight tended to be less (*P* = 0.08) for NR dams than CON. As a percentage of total weight or number, ipsilateral dry cotyledonary weight, intercotyledonary weight, placental weight, and number of cotyledons were not affected (*P *≥ 0.38) by nutritional plane.

**Table 3.** Effects of late gestational nutritional plane of primiparous beef females on placental size

	Nutritional plane^1^			
Item	CON	NR	SEM^2^	*P*-value
Ipsilateral side				
Dry cotyledonary weight, g	54.4	44.8	5.5	0.23
Dry intercotyledonary weight, g	96.6	84.2	8.2	0.28
Dry placental weight, g	156	129	12	0.12
Number of cotyledons	52.9	59.1	1.3	0.003
Average dry cotyledon weight, g	1.04	0.79	0.10	0.08
Dry cotyledonary:intercotyledonary weight	0.619	0.550	0.047	0.30
Dry cotyledonary weight^3^, % of total	81.0	79.9	3.0	0.80
Dry intercotyledonary weight^3^, % of total	80.8	80.7	2.1	0.98
Dry placental weight^3^, % of total	81.0	80.5	2.3	0.88
Number of cotyledons^3^, % of total	61.3	56.5	3.8	0.38
Contralateral side				
Dry cotyledonary weight, g	13.2	11.4	2.4	0.60
Dry intercotyledonary weight, g	21.9	20.3	3.0	0.70
Dry placental weight, g	35.1	31.7	4.9	0.62
Number of cotyledons	34.9	45.8	4.4	0.09
Average dry cotyledon weight, g	0.335	0.248	0.052	0.25
Dry cotyledonary:intercotyledonary weight	0.618	0.565	0.097	0.70
Whole placenta				
Dry cotyledonary weight, g	69.0	56.4	6.4	0.18
Dry intercotyledonary weight, g	120	105	10	0.26
Dry total placental weight, g	193	161	14	0.10
Number of cotyledons	89	105	5	0.03
Average dry cotyledon weight, g	0.803	0.550	0.081	0.04
Dry cotyledonary:intercotyledonary weight	0.615	0.554	0.044	0.32
Placental efficiency^4^, kg calf birth weight/g total placental DM	0.144	0.146	0.009	0.89
Placental weight relative to dam BW^5^, g total placental DM/kg dam BW	0.425	0.405	0.032	0.65
Average umbilical vessel diameter, mm	7.15	6.74	0.22	0.20

^1^Primiparous dams were individually-fed either 100% (Control; CON) or 70% (Nutrient Restricted; NR) of estimated metabolizable energy and metabolizable protein requirements for maintenance, pregnancy, and growth from day 160 of gestation to parturition.

^2^Standard error of the mean for CON (*n* = 8 or 9) and NR (*n* = 10).

^3^Ipsilateral side as percentage of whole placenta.

^4^Calf birth weight determined at 1.0 ± 0.3 h (SD) of age (pre-suckling).

^5^Dam body weight determined on days 1 and 2 post-calving.

Nutritional plane did not affect (*P* ≥ 0.60; Table 3) contralateral dry cotyledonary, intercotyledonary, and placental weights. Contralateral number of cotyledons tended to be greater (*P* = 0.09) for NR dams than CON, but average dry cotyledon weight and cotyledonary:intercotyledonary weight were not affected (*P* ≥ 0.25) by nutritional plane.

Whole dry cotyledonary and intercotyledonary weights were not affected (*P* ≥ 0.18; Table 3) by nutritional plane, but whole dry total placental weight tended to be less (*P* = 0.10) for NR dams than CON. Whole placental number of cotyledons was greater (*P* = 0.03), but average dry cotyledon weight was less (*P* = 0.04) for NR dams than CON. Whole placental dry cotyledonary:intercotyledonary weight, placental efficiency, placental weight relative to dam BW, and average umbilical vessel diameter were not affected (*P* ≥ 0.20) by nutritional plane.

Time from parturition to cotyledonary tissue sampling tended to be greater (*P* = 0.07; Table 4) for NR dams than CON. Cotyledonary relative mRNA expression of GLUT1 was greater (*P* = 0.04) and SNAT2 tended to be greater (*P* = 0.07) for NR dams than CON. Late gestational nutritional plane did not affect (*P* ≥ 0.13) cotyledonary relative mRNA expression of GLUT3, GLUT4, GLUT5, 4F2hc, CAT1, LAT1, LAT2, VEGFA, FLT1, KDR, NOS3, GUCY1B3, or PAG2.

**Table 4.** Effects of late gestational nutritional plane of primiparous beef females on cotyledonary relative mRNA expression^1^

	Nutritional plane^2^			
Item	CON	NR	SEM^3^	*P*-value
Time from parturition to cotyledonary tissue sampling, h	3.71	4.80	0.41	0.07
Glucose transporters				
GLUT1 (SLC2A1)	0.44	0.60	0.05	0.04
GLUT3 (SLC2A3)	0.76	0.88	0.06	0.16
GLUT4 (SLC2A4)	0.88	1.13	0.40	0.67
GLUT5 (SLC2A5)	0.45	0.59	0.06	0.17
Amino acid transporters				
4F2hc (SLC3A2)	1.22	1.13	0.15	0.69
CAT1 (SLC7A1)	0.83	1.44	0.35	0.25
LAT1 (SLC7A5)	0.99	0.76	0.11	0.15
LAT2 (SLC7A8)	0.91	0.90	0.08	0.90
SNAT2 (SLC38A2)	0.82	1.08	0.09	0.07
Angiogenic factors				
VEGFA	0.80	0.66	0.11	0.40
FLT1 (VEGFA receptor 1)	0.88	1.06	0.21	0.56
KDR (VEGFA receptor 2)	0.77	1.17	0.24	0.27
NOS3	0.93	1.21	0.41	0.64
GUCY1B3 (nitric oxide receptor)	0.60	1.09	0.21	0.13
PAG2	0.50	0.55	0.07	0.59

^1^Target mRNA cycle threshold values were normalized to G3PDH (reference gene) with mRNA expression calculated relative to a pooled internal control using the 2^−ΔΔCT^ method.

^2^Primiparous dams were individually-fed either 100% (Control; CON) or 70% (Nutrient Restricted; NR) of estimated metabolizable energy and metabolizable protein requirements for maintenance, pregnancy, and growth from day 160 of gestation to parturition.

^3^Standard error of the mean for CON (*n* = 9) and NR (*n* = 9 or 10).

### Calving characteristics and calf size at birth

Gestation length was not affected (*P* = 0.53; Table 5) by late gestational nutritional plane. The percentage of heifers assisted at calving was not affected (*P* = 0.57) by nutritional plane, and no calves born to NR or CON dams were abnormally presented. The 2 CON and 1 NR dam that were assisted at calving were manually assisted deliveries.

**Table 5.** Effects of late gestational nutritional plane of primiparous beef females on beef calf size at birth

	Nutritional Plane^1^			
Item	CON	NR	SEM^2^	*P*-value
Gestation length, d	274	273	1	0.53
Calf sex, % male	60.0	41.7	-	-
Assisted calving, %	20.0	8.33	-	0.57
Abnormal presentation, %	0.0	0.0	-	-
Calf size				
Birth weight^3^, kg	26.3	22.5	1.2	0.03
Shoulder to rump length, cm	53.2	51.3	0.9	0.17
Heart girth, cm	68.8	63.9	1.3	0.02
Abdominal girth, cm	64.4	59.3	1.8	0.06
Flank girth, cm	59.6	55.2	1.9	0.11
Cannon circumference, cm	11.5	11.0	0.2	0.09
Cannon length, cm	16.7	15.9	0.5	0.20
Coronet circumference, cm	17.6	16.6	0.3	0.04
Height at shoulder, cm	64.4	63.0	0.9	0.29
Calf ponderal index^4^, kg/m^3^	174	166	6	0.30
Heart girth:length^5^	1.29	1.25	0.02	0.17
Volume^6^, L	17.8	14.6	1.0	0.03
Longissimus muscle area^7^, cm^2^	6.83	5.62	0.44	0.06
Birth weight^8^, % initial dam BW	5.68	4.80	0.26	0.03
Birth weight^9^, % final dam BW	5.72	5.68	0.32	0.94

^1^Primiparous dams were individually-fed either 100% (Control; CON) or 70% (Nutrient Restricted; NR) of metabolizable energy and metabolizable protein requirements for maintenance, pregnancy, and growth from day 160 of gestation to parturition.

^2^Standard error of the mean for CON (*n* = 10) and NR (*n* = 12).

^3^Calf birth weight determined at 1.0 ± 0.3 h (SD) of age (pre-suckling).

^4^Ponderal index = calf birth weight (kg) / shoulder to rump length (m)^3^.

^5^Ratio of heart girth (cm):shoulder to rump length (cm).

^6^Volume (L) = [π × average girth radius (cm)^2^ × shoulder to rump length (cm)] / 1,000.

^7^Determined at 2 d of age.

^8^Dam body weight before dietary treatment initiation (days 158 and 159 of gestation).

^9^Dam body weight determined on days 1 and 2 post-calving.

Calf birth weight was 14.4% less (*P* = 0.03; Table 5) for NR dams than CON (for those with successful placenta collections: *P* = 0.09; 23.1 vs. 26.2 ± 1.3 kg). Additionally, calves born to NR dams had smaller (*P* ≤ 0.04) heart girth, coronet circumference, and volume and tended to have smaller (*P* ≤ 0.09) abdominal girth, cannon circumference, and longissimus muscle area than calves born to CON. Calf shoulder to rump length, flank girth, cannon length, height at shoulder, ponderal index, and heart girth:length were not affected (*P *≥ 0.11) by nutritional plane. Calf birth weight as a percent of dam BW on day 158 of gestation was less (*P* = 0.03) for calves born to NR dams compared with calves born to CON; however, calf birth weight as a percent of dam post-calving BW was not affected (*P* = 0.94) by nutritional plane.

## Discussion

The experiment reported here and the experiment reported in Redifer et al. (2023, 2024) used the same late gestational nutrient restriction model but 2 different groups of pregnant beef heifers. The studies were conducted in back-to-back years to facilitate offspring data collection at different experimental end points (48 h postnatal vs. weaning). The single, overarching hypothesis was that late gestational nutrient restriction would impair uteroplacental nutrient transport, resulting in intrauterine growth restriction. We anticipated this would be observed in both experiments given the same nutrient restriction model, dam parity, diets, facilities, and season of calving were used. Pregnant females in both studies originated from the same University of Missouri fall-calving beef herd, but the heifers’ sires and the sire of the offspring differed between experiments. Pregnant females were housed in the same Calan gate facility during both experiments, but identical climatic conditions were not experienced between years. Despite controlling most confounding factors, we observed conflicting results in these 2 experiments, which is similar to conflicting results found in aggregate across the literature: calves born to NR dams weighed 14.4% less at birth in the current experiment, but fetal growth was unchanged in the previous experiment (Redifer et al., 2023). These 2 experiments with differing fetal growth results from the same research model allow for a comparison of the physiological mechanisms that likely resulted in these divergent outcomes of late gestational nutrient restriction in these and other studies. First-parity beef females that were NR during late gestation were 62.1 kg and 2.1 BCS less than CON females post-calving in the current study, which was nearly identical to the 63.6 kg and 2.0 BCS difference observed post-calving in Redifer et al. (2023). Overall, although negative effects of nutrient restriction on maternal BW, body composition, and metabolism were consistent in both studies, placental adaptations without uterine blood flow changes appeared to drive fetal growth differences observed.

### Uteroplacental nutrient transport capacity

Uterine blood flow was not affected by late gestational nutrient restriction in the current study or Redifer et al. (2024). We previously reported positive relationships among late gestational total uterine artery blood flow, dry total placental weight, and calf birth weight (Redifer and Meyer, 2020; Redifer et al., 2021); thus, it was surprising that placental size and birth weight decreased independent of a reduction in uterine blood flow. Together, this study and Redifer et al. (2024) strongly suggest that uterine blood flow is resistant to changes in maternal nutritional status in late pregnant fall-calving beef heifers. The variability in late gestational total uterine blood flow in cattle is noteworthy (Herzog et al., 2011). To minimize controllable variation, we measured uterine blood flow pre-treatment to ensure it was similar prior to nutrient restriction, utilized similar dam and fetal genetics, had a single technician each for ultrasound and measurements, sampled during a consistent time range, and maintained angle of insonation in a narrow range. Past reports on the effects of late gestational undernutrition on uterine blood flow have been inconclusive (reviewed in Redifer et al., 2024), even finding conflicting results within laboratories or experimental designs, but often angle of insonation and pre-treatment blood flow were not considered or reported.

Maternal heart rate increased for CON females but decreased for NR females from days 157 to 181 of gestation and was between 10.2% and 14.9% less for NR females for the remainder of pregnancy, which strongly resembles the findings in Redifer et al. (2024) and Kennedy et al. (2016b). These results collectively support the hypothesis of Thomas and Moore (1951) that maternal heart rate follows changes in nutritional plane. In other studies where maternal heart rate was not affected by nutritional plane, performance of mature beef cows fed only poor-quality forage did not diverge as dramatically from supplemented females (Mordhorst et al., 2017; Tanner et al., 2023).

Changes over time in ipsilateral and contralateral uterine artery hemodynamics are largely in agreement with the seminal papers of Bollwein et al. (2002) and Panarace et al. (2006), with patterns and values comparable to Redifer et al. (2024). Ipsilateral peak systolic velocity and mean velocity remained stable for NR females from days 223 to 244 of gestation but decreased for CON, which was unexpected and differed from these previous reports. Uterine blood flow determination for day 265 was suspended early in sampling after short gestation lengths were observed, so we can only speculate if ipsilateral velocities would have remained divergent for nutritional planes or if the difference on day 244 was an anomaly.

For the subset of dams whose expelled placentas were successfully collected, calf birth weight was 11.8% less and dry total placental weight was 16.6% less for NR dams than CON. Placental mass reduction in NR females was not caused by asymmetrical growth within one side (ipsilateral vs. contralateral) or tissue type (cotyledonary vs. intercotyledonary). This is in contrast with Redifer et al. (2024), where NR dams had less contralateral placental growth, but whole placental weight was not affected. Placental efficiency (kg calf birth weight/g total placental DM) was not affected by nutritional plane in either study and had similar values, despite the disparity in calf birth weight outcomes, which suggests that the insult on placental size in the current study directly contributed to the depression in calf birth weight for NR dams. In other past beef cattle studies, placental size was not affected by late gestational undernutrition (Kennedy et al., 2016b; Mordhorst et al., 2017; Hummel et al., 2021), but had been affected by peripartum BCS (Redifer et al., 2021), regardless of calf birth weight outcomes.

Ipsilateral placental weights as a proportion of total closely resembled ipsilateral blood flow as a proportion of total and were in a similar range as the values reported in Redifer et al. (2024). In the current study, NR dams had 31.5% smaller average cotyledon size, which resulted from both decreased cotyledonary mass and increased cotyledon number. All functional placentomes are believed to already be established in early gestation (Laven and Peters, 2001; Assis Neto et al., 2010), so late gestational nutrient restriction altering, and especially increasing, cotyledon number was unexpected and likely is an uncontrollable discrepancy that existed prior to our nutritional treatments.

Nutrient restricted dams had 36.4% greater cotyledonary mRNA expression of GLUT1 and 31.7% greater expression of SNAT2, but other amino acid transporters, glucose/fructose transporters, angiogenic factors, and PAG2 were not affected. In our previous study, placentas of NR dams had greater GLUT3, GLUT4, and NOS3 cotyledonary transcripts in addition to greater GLUT1 and SNAT2 transcripts, likely as a compensatory mechanism that contributed to spared fetal and placental growth (Redifer et al., 2024). Other studies have yielded inconsistent findings, with late gestational undernutrition not affecting or increasing glucose and amino acid transporter expression in some instances (Picking et al., 2020; Swanson et al., 2022) but decreasing nutrient transporter expression in others (Kang et al., 2022). Similarly, angiogenic factor expression was decreased by nutrient restriction in Contreras-Correa et al. (2022) but not altered in Picking et al. (2020). Previously, late gestational nutritional plane did not affect cotyledonary mRNA expression of PAG2 (Redifer et al., 2024) or placentomal PAG1 transcripts (Picking et al., 2020), which concurs with the current findings. Discrepancies among results discussed here may involve whether cotyledonary or whole placentomal tissues were collected as well as if pregnant dams were slaughtered, pre-term cesarean-sections were performed, or dams went through parturition and placental expulsion naturally.

Time from parturition to cotyledonary tissue sampling ranged from 2.2 to 6.4 h for animals with adequate cotyledonary RNA integrity number. The delayed time to cotyledonary tissue sampling for NR dams, which closely represents time to placental expulsion, could have affected mRNA transcript abundance; thus, it was included as a covariate in the statistical models. Previously, late gestational nutritional plane did not affect time from parturition to cotyledonary tissue sampling (Redifer et al., 2024) or incidence of retained placentas (Tudor, 1972). It is worth noting that in current study, beef females had their calves removed immediately after birth and colostrum hand-milked at 12-h intervals, which may have disturbed normal postpartum endocrine and behavioral changes involved in placental expulsion (Attupuram et al., 2016).

### Fetal growth

The reduction in fetal growth (14.4%) mirrored maternal BW differences post-calving (13.5%), and as a result calf birth weight relative to maternal BW after 120 d of nutrient restriction was not affected by nutritional plane. Although nutrient restriction during late gestation decreased calf birth weight in the current study, fetal growth was not affected using the same model in Redifer et al. (2023). Past studies evaluating late gestational undernutrition in beef cattle were also nearly equally split in those that observed a reduction in calf birth weight and those where calf birth weight was similar to adequately-fed dams (reviewed in Redifer et al., 2023). Late gestational undernutrition has also decreased pre-term fetal weight in Contreras-Correa et al. (2021), but inconsistently (Long et al., 2021b). Similar to our conflicting results, pregnant recipient heifers were fed to term and the nutrient restricted dams had smaller calves at birth than adequately-fed dams in Long et al. (2021a), which used the same nutrient restriction model as Long et al. (2021b). These collectively imply that the beef female is resilient to late gestational undernutrition up to a point, but there is a threshold of maternal nutrient balance after which fetal and placental outcomes are compromised. Furthermore, the inconsistency of reduced fetal growth within nutrient restriction models suggests that this threshold is not the same for all females.

Soft tissue and muscle growth appear to be a lower priority than skeletal growth when faced with decreased nutrient availability during late gestation, as girth measures, longissimus muscle area, and volume were more negatively impacted than body length and shoulder height. Calf body length was reduced in Kroker and Cummins (1979) but not in Laster (1974), despite calf birth weight being decreased in both studies due to late gestational undernutrition. Laster (1974) hypothesized that decreased fetal growth was attributed to reduced soft tissue growth, which would agree with girth measures being more severely affected than skeletal size measures in the current study.

Gestation length can have a major influence on calf birth weight (Holland and Odde, 1992), but it does not account for the birth weight depression of NR females in the current study. Average gestation length of 274 d (range: 262 to 280 d) for all females was shorter than what is expected for current U.S. beef genetics. By regressing calf birth weight on gestation length, an increase of 1 d in gestation length increased calf birth weight by 0.75 kg for CON females. If females in the current study had calved at a similar gestation length as those in Redifer et al. (2023), birth weight of calves born to CON dams would have been similar in both studies. Others have reported shortened gestation lengths in nutrient restricted females that contributed to birth weight reductions as severe as 20% to 25% (Tudor, 1972; Kroker and Cummins, 1979).

The percentage of females assisted at calving was not affected by nutritional plane, and all 3 incidences of dystocia were resolved by manual traction. Absolute calf birth weight and calf birth weight relative to dam BW at parturition were low for females on both nutritional planes, which likely contributed to the low frequency of calving difficulties. No abnormal presentations were observed in the current study, which is contrary to Kroker and Cummins (1979) and Redifer et al. (2023), where late gestational nutrient restriction in heifers increased malpresentations at birth.

### Maternal body composition and metabolism

The last third of pregnancy represents the catabolic phase, when gestating females will mobilize maternal stores that were built up earlier in pregnancy to satisfy nutrient requirements of the developing fetus, especially when nutrient availability is limited (Naismith and Morgan, 1976; Robinson, 1986). Post-calving, non-gravid BW of NR females was 64.9 kg less and BCS was 2.0 less than at treatment initiation. Additionally, late gestational maternal BCS and backfat thickness losses for NR dams were in good agreement, being 38.8% and 33.6% less, respectively, than CON post-calving.

When animals are required to draw from their own body stores for nutrients, adipose tissue is often mobilized more immediately than muscle tissue (Chilliard et al., 1998). Surprisingly, maternal longissimus muscle area was less in NR dams after 42 d of nutrient restriction, but maternal backfat thickness differences were not observed until 84 d of underfeeding. Additionally, CON females maintained BCS and backfat thickness throughout late gestation, but longissimus muscle area decreased. Primiparous beef females fed adequately during late gestation maintained longissimus muscle area in Long et al. (2021a, 2021b), while nutrient restriction decreased longissimus muscle area at day 270 of gestation only in Long et al. (2021a). NR dams achieved similar skeletal growth as CON in the current study, as shoulder height was not affected by nutritional plane despite maternal BW decreasing 0.57 kg/d for NR dams during this period. Redifer et al. (2023) did not consider how late gestational undernutrition affected shoulder height or longissimus muscle area, so it is unknown if these findings were consistent with our previous study.

As expected, NR dams had less glucose, urea N, and triglycerides in maternal circulation during late gestation, but NEFA were elevated. Circulating metabolites responded to late gestational undernutrition in a similar fashion as in Redifer et al. (2023), but relative differences between nutritional planes were not as dramatic within timepoints or as consistent across days. In both Redifer et al. (2023) and the current study, NEFA concentration decreased for NR dams after prolonged undernutrition, and urea N concentration increased from days 244 to 265 for both nutritional planes. After extended nutrient restriction, NEFA concentrations will plateau and even decrease as lipolytic activity lessens to spare basal adipose stores (Chilliard et al., 1998). Dietary provision of CP did not change, or slightly decreased, after day 244 of gestation for all females, which suggests the increase in urea N until day 265 of gestation may be a result of urea N returning to maternal circulation from fetal circulation as a waste product of uteroplacental deamination of amino acids for energetic needs (Ferrell et al., 1983). Another possibility is that the increase in urea N was a result of catabolism of maternal muscle tissue, as evident by the decrease in longissimus muscle area for all females near the end of pregnancy.

### Differences in maternal nutrient use

Despite altering supplement formulation when CON BW gain was not as expected, CON females in the current study underperformed relative to predicted performance. While the relative difference in maternal post-calving BW and body composition between nutritional planes was similar to Redifer et al. (2023), CON females in the current study did not achieve the projected maternal average daily gain (0.36 kg/d). In fact, CON dam maternal average daily gain (0.05 kg/d) was not different than zero, which was unexpected. Per NASEM (2016), the target calving weight for primiparous females is 80% of the estimated mature cow weight. Estimated mature cow BW for these females was 613 kg; thus, CON female post-calving BW (460 kg) was 75% of estimated mature BW despite less than expected gain during late gestation. It is unknown if females in the current study entered late gestation at a different relative maturity or overall body composition than those in Redifer et al. (2023).

Calf birth weight for CON females was less than the expected birth weight of 34.0 kg used for formulating nutrient requirements of pregnancy, even if gestation lengths had been more normal. This means that if NASEM (2016) nutrient recommendations for pregnancy are correct, more nutrients were available for fetal growth than appear to have been used given the birth weights observed in CON; thus, nutrients in surplus should have hypothetically been partitioned to growth and deposition of excess body condition, but this did not occur.

The marginal maternal growth and smaller than anticipated calf birth weights beg the question of why the CON animals responded so poorly to provision of current nutrient recommendations. Because CON females in the current study and in Redifer et al. (2023) were fed similarly but different responses were observed, it appears CON females in the current study were less efficient in their use of nutrients than those in Redifer et al. (2023). This may have been due to differences in maintenance energy expenditures or nutrient utilization for fetal and maternal growth. It is also plausible that current nutrient recommendations underestimate the maintenance costs and direct nutrient needs of gestation, but the females in Redifer et al. (2023) happened to have similar nutrient needs to those underestimated recommendations. If this is true, CON females in the current experiment were in fact fed less than 100% of their true requirements, and NR females were fed less than 70% of their requirements. In that case, it is possible that the more severe plane of undernutrition than intended resulted in the observed intrauterine growth restriction in the current study. More research is needed to determine if the seminal data and equations utilized in the current nutrient recommendations of pregnancy still fit modern U.S. beef genetics or if nutrient recommendations for pregnant beef heifers and cows need to be reevaluated.

Forage used in this study was from the same lot as Redifer et al. (2023), and storage allowed for minimal decomposition. Although hay DMI was not affected by nutritional plane, it decreased by an average of 31.9% from the week beginning on day 188 of gestation to parturition, which may have been influenced by lower overall diet CP. This dramatic decline in hay DMI was not observed in Redifer et al. (2023), and it made formulation to meet targeted nutrient intakes more difficult. Forage energy concentrations are difficult to estimate; thus, it is also possible that hay ME was overestimated in these experiments, resulting in the observed performance for this less efficient group of females.

## Conclusions

First-parity beef females that were NR during late gestation lost maternal BW and mobilized adipose and muscle tissue stores but maintained skeletal growth compared with adequately-fed females. Nutrient restricted females had reduced metabolites in maternal circulation but cotyledonary transcripts of the glucose transporter GLUT1 and amino acid transporter SNAT2 were upregulated. Whole dry placental weight was smaller for NR females despite total uterine artery blood flow being resistant to nutrient restriction and remaining similar to CON females. The insults to nutrient availability in maternal circulation and placental size were enough to compromise uteroplacental nutrient transport capacity, and calves born to NR females were 14.4% smaller than calves born to CON. In a past study using this experimental model, NR females sacrificed maternal growth to spare fetal growth, and uteroplacental measures were not negatively affected (Redifer et al., 2023, 2024). The varied response and how one cohort was resilient to gestational undernutrition but another cohort was vulnerable to deleterious placental and fetal effects deserves further investigation.

## Abbreviations

AI: artificial insemination
BCS: body condition score
BW: body weight
CIDR: controlled internal drug release
CP: crude protein
DDGS: dried distillers grains with solubles
DM: dry matter
DMI: dry matter intake
ME: metabolizable energy
MP: metabolizable protein
mRNA: messenger RNA
NEFA: non-esterified fatty acids
RNA: ribonucleic acid
